# Current Addiction in Youth: Online Sports Betting

**DOI:** 10.3389/fpsyt.2020.590554

**Published:** 2021-01-13

**Authors:** Núria Aragay, Laia Pijuan, Àngela Cabestany, Irene Ramos-Grille, Gemma Garrido, Vicenç Vallès, Esther Jovell-Fernández

**Affiliations:** ^1^Pathological Gambling Unit, Department of Mental Health, Consorci Sanitari de Terrassa, Terrassa, Spain; ^2^Faculty of Medicine and Health Sciences, Universitat Internacional de Catalunya, Barcelona, Spain; ^3^Department of Mental Health, Consorci Sanitari de Terrassa, Terrassa, Spain; ^4^Department of Clinical and Health Psychology, Universitat Autònoma de Barcelona, Barcelona, Spain; ^5^Community Mental Health Service, Department of Mental Health, Consorci Sanitari de Terrassa, Terrassa, Spain; ^6^Department of Epidemiology, Consorci Sanitari de Terrassa, Terrassa, Spain

**Keywords:** gambling disorder, online gambling, sports betting, offline gambling, slot machine, predictors

## Abstract

**Background:** Gambling landscape has changed in recent years with the emergence of online gambling (OG). Greater accessibility and availability of this betting modality can increase the risk of developing a gambling disorder (GD). Online sports betting (OSB) is currently the most common type of OG, but little is known about the clinical characteristics of OSB compared to slot-machine (SM) gamblers, the most common offline gambling disorder.

**Methods:** This was a prospective study conducted between October 2005 and September 2019, and included outpatients diagnosed with GD seen in a Pathological Gambling and Behavioral Addictions referral unit. Only patients with OSB and SM disorders were included. The main objective was to assess the clinical profile of OSB compared to SM gamblers, and to define clinical predictors for developing OSB gambling disorder. Logistic regression was performed to determine the effects of variables on the likelihood of this disorder.

**Results:** Among 1,186 patients attended in our Unit during the study period, 873 patients were included; 32 (3.7%) were OSB gamblers and 841 (96.3%) were SM gamblers. Overall, mean age was 45 ± 13 years and 94.3% were men. Compared to SM patients, OSB patients were younger (34.9 ± 9.5 vs. 45.3 ±13), more frequently single (43.8 vs. 20.6%) and had a university education level (43.8 vs. 4.5%); they were also more frequently non-smokers (18.7 vs. 66.7%) and had fewer psychiatric comorbidities (12.5 vs. 29.4%) than SM gamblers. GD duration before treatment initiation was shorter in OSB patients than in SM gamblers, most of them (81.3 vs. 42.4%) with ≤ 5 years of GD duration. OSB gamblers showed significant differences in weekly gambling expenditure, spending higher amounts than SM patients. Younger age (OR: 0.919; 95% CI: 0.874–0.966), university education level (OR: 10.658; 95% CI: 3.330–34.119), weekly expenditure >100€ (OR: 5.811; 95% CI:1.544–21.869), and being a non-smoker (OR:13.248; 95% CI:4.332–40.517) were associated with an increased likelihood of OSB gambling behavior.

**Conclusions:** We identified different profiles for OSB and SM gamblers. Younger age, university education level, higher weekly expenditure, and non-smoking habit were associated with OSB compared to SM disorders. Prevention strategies should help young people become aware of the severe risks of OSB.

## Introduction

Gambling disorder (GD) is a gambler's inability to control their gambling behavior despite the negative consequences that this entails. The latest version of the Diagnostic and Statistical Manual of Mental Disorders, Fifth Edition (DSM-5), includes this disorder within the “Addictive and substance-related disorders,” and describes it as “a maladaptive, persistent and recurrent behavior that disrupts personal, family and/or work” (1).

In a systematic review of 69 studies from different countries, adult gambling prevalence was between 0.7 and 6.5% (2). However, most of these epidemiological studies were based on offline gambling samples. Evidence on online gambling practices is scarce, but prevalence is estimated to range from 1 to 13% of the general population (3, 4). In a study conducted in Spain, Choliz et al. found a prevalence of 0.56% in adults, and 1.04% in young people (5).

Gambling was legalized in Spain in 1977. Slot-machines (SM) appeared in 1981, and rapidly became one of the most widely used forms of gambling, and the cause of most gambling problems (6).

The gambling market has changed in recent years due to the emergence of new technologies and online gambling (OG) (6, 7). The possibility of gambling from home and betting with “virtual money” has increased the accessibility, frequency, disinhibition, and lack of control of OG (8). All these features, as well as the diversification in different types of online games, can increase the risk of developing problems derived from OG (9). There are different types of OG, such as sports betting, poker, casino games, bingo, and gambling machines, but online sports betting (OSB) is currently the most common OG modality.

The advertising and marketing strategies used by the online gaming sector provide an unreal image of OSB as a lucrative leisure activity that can bring economic and social success to the gambler. It establishes a relationship between fun, sports, competition, friendship, and other values associated with adolescence and youth. All these characteristics have contributed to a better positive social perception of OSB (9, 10).

Some previous studies have compared general samples of online and offline gamblers (11–13). However, little is known about specific comparisons between OSB and land-based SM gamblers. In fact, these were the most common gambling modes in 2019 in Spain (31 and 21%, respectively) (14). Because of their high prevalence, particularly among young people, these forms of gambling are an important health problem that must be addressed and prevented (7). So we aimed to compare gambling behavior characteristics between OSB and SM gamblers, and to define clinical predictors for OSB.

## Materials and Methods

### Study Design

This was a prospective study conducted from October 2005 to September 2019 among outpatients seen in a Pathological Gambling and Behavioral Addictions Unit from a referral population of 1.3 million. Most patients are referred from primary care physicians within the public healthcare system.

All patients were diagnosed with GD according to the DSM-IV-TR, or DSM-5 when appropriate (1, 15). For this study, only patients with OSB and SM disorders were included. All participants provided written or oral informed consent. The study was conducted in accordance with the Declaration of Helsinki and was approved by the Ethics Committee of Consorci Sanitari de Terrassa (Barcelona, Spain). All patients were treated and followed-up by a team of psychologists, supervised by a senior clinical psychologist with more than 15 years' experience in the diagnosis and treatment of GD.

The therapeutic program has been described elsewhere and consists of individualized outpatient cognitive-behavioral therapy for PG, aimed at achieving abstinence from gambling. Treatment was protocolized, and the main techniques used were psychoeducation, motivational interviewing, stimulus control, cognitive restructuring and relapse prevention (16, 17).

The main objective was to assess the clinical profile of OSB gamblers compared with SM gamblers, and to define clinical predictors for developing OSB.

### Variables

#### Gambling Variables

We recorded the type of game (OSB or SM), age of gambling behavior onset, duration of GD, frequency of gambling, and weekly gambling expenditure.

#### Sociodemographic and Clinical Variables

We recorded age, gender, marital status, education level, and employment status. Psychiatric comorbidities were assessed according to DSM-IV-TR or DMS-5 (affective disorder, psychotic disorder, anxiety disorder, adaptive disorder, attention deficit disorder, and substance use disorder).

#### Statistical Analysis

A descriptive statistical analysis was performed for all categorical and continuous variables and expressed as proportions or means with standard deviations (SD), respectively. We used chi-square or Fisher's exact tests to compare categorical data between groups. Continuous variables were compared using the Student *t*-test. We used two-tailed unpaired *t*-tests to compare normally distributed continuous data between two groups, and the Mann-Whitney U test for non-normally distributed continuous data comparisons. To control the effect of age on the differences found between OSB and SM gamblers, we performed a *post-hoc* analysis including gamblers who started gambling at ≤ 25 years of age. *P*-values < 0.05 were considered statistically significant.

Logistic regression analyses were performed to identify associated risk factors for OSB gambling and presented as odds ratios (OR) with 95% confidence intervals (95% CI). For the manual backward stepwise multivariate logistic regression model, we assessed variables that had a significant p level < 0.05 in univariate analyses. The Hosmer and Lemeshow test was applied; a *p*-value of 0.05 or higher indicated that the model fitted well with the data. The variance inflation factors (VIF) of each variable included in the final model were computed, and a VIF of >10 indicated that multicollinearity of the corresponding variable was high. Analyses were performed using SPSS, version 25 for PC (SPSS Inc., Chicago, IL, USA).

## Results

Among 1,186 patients attended in our Unit during the study period, 172 patients were excluded due to other behavioral addictions and 141 patients due to other types of gambling. Finally, 873 patients were included: 32 (3.7%) were OSB gamblers and 841 (96.3%) were SM gamblers. Overall, mean age was 45 ± 13 years and 94.3% were men. Most patients had a stable partner (54.5%), had completed primary or secondary education (83.2%), were employed (53%) and were smokers (65%). Mean age for gambling onset was 26.5 ± 10.9 years. Most patients (55.3%) had a gambling history of >5 years.

Compared to SM patients, OSB patients were younger (34.9 ± 9.5 vs. 45.3 ± 13), more frequently single (43.8 vs. 20.6%) and with university education level (43.8 vs. 4.5%); they were also more frequently non-smokers (18.8 vs. 66.7%) and had fewer psychiatric comorbidities (12.5 vs. 29.4%) than SM gamblers. Duration of the GD was shorter in OSB patients than in SM gamblers, most of them (81.3 vs. 42.4%) with ≤ 5 years of GD duration before treatment initiation. OSB gamblers showed significant differences in their weekly gambling expenditure, spending higher amounts than SM patients. Comparison between OSB and SM gamblers is shown in Table 1.

**Table 1:** Bivariate analysis comparing online sports betting and slot machine gamblers.

**Variable**	**Online sports** **betting gamblers** **(*n* = 32)**	**Slot machine** **gamblers** **(*n* = 841)**	***p*-value**
Age, mean (SD)	34.9 ± 9.5	45.3 ± 13.0	**0.000**
**Gender**, ***n*** **(%)**			
Male	31 (96.9%)	792 (94.2%)	1.000
Female	1 (3.1)	49 (5.8%)	
**Marital status**, ***n*** **(%)**			
Single	14 (43.8%)	173 (20.6%)	**0.002**
With partner	18 (56.3%)	668 (79.4%)	
Stable partner	14 (43.8%)	462 (54.9%)	
Separated	2 (6.3%)	129 (15.3%)	
Divorced	2 (6.3%)	64 (7.6%)	
Widowed	0	13 (1.5%)	
**Education level**, ***n*** **(%)**			
University	14 (43.8%)	38 (4.5%)	**0.000**
Non-university	18 (56.3%)	776 (92.3%)	
Primary/Secondary	18(56.3%)	708 (84.2%)	
Illiterate	0	68 (8.1%)	
**Employment status**, ***n*** **(%)**			
Employed	22 (68.8%)	441 (52.4%)	0.077
Unemployed	10 (31.3%)	393 (46.7%)	
Student	5 (15.6%)	2 (0.2%)	
Age at gambling onset, mean (SD)	26.41 ± 9.5	26.52 ± 11	0.956
**Duration of GD before treatment**, ***n*** **(%)**			
≤ 5 years	26 (81.3%)	357 (42.4%)	**0.000**
>5 years	6 (18.8%)	477 (56.7%)	
6–10 years	5 (15.6%)	178 (21.2%)	
>10 years	1 (3.1%)	299 (35.6%)	
Daily frequency of gambling, *n* (%)	16 (50%)	344 (41%)	0.204
**Gambling expenditure per week**, ***n*** **(%)**			
≤ 100€/week	6 (18.8%)	377 (44.8%)	**0.003**
>100€/week	22 (68.8%)	372 (44.2%)	
>500€/week	8 (25%)	82 (9.8%)	
**Psychiatric comorbidity**, ***n*** **(%)**	4 (12.5%)	247 (29.4%)	**0.039**
Affective disorder	0 (0%)	44 (5.2%)	0.400
Psychotic disorder	0 (0%)	48 (5.7%)	0.251
Anxiety disorder	1 (3.1%)	24 (2.9%)	0.612
Adaptive disorder	1 (3.1%)	40 (4.8%)	1.000
Attention deficit disorder	1 (3.1%)	17 (2%)	0.493
**Substance use disorder**, ***n*** **(%)**			
Alcohol dependence	3 (9.4%)	186 (22.1%)	0.200
Tobacco dependence	6 (18.8%)	561 (66.7%)	**0.000**
Cannabis dependence	0	56 (6.7%)	0.400
Cocaine dependence	0	42 (5%)	0.629

*The blod values means “statistically significant values”*.

We aimed to assess the impact of OSB compared to SM among those who started gambling when young. We performed a *post-hoc* analysis in the subgroup of gamblers who started gambling at ≤ 25 years old. Compared to SM gamblers, OSB gamblers were more frequently single (72.2 vs. 26%) and with university education level (50 vs. 4.8%). In addition, OSB gamblers had higher weekly gambling expenditure and shorter length of GD before starting treatment compared to SM gamblers (88.9% of OSB vs. 41.2% of SM gamblers reported GD onset ≤ 5 years) (Table 2).

**Table 2:** Bivariate analysis comparing online sports betting and slot machine gamblers with gambling onset before 25 years of age.

**Variable**	**Online sports** **betting gamblers** **(*n* = 18)**	**Slot machine** **gamblers** **(*n* = 438)**	***p*-value**
Age, mean (SD)	29.72 ± 7.6	40.07 ± 10.9	**0.000**
**Gender**, ***n*** **(%)**			
Male	17 (94.4%)	426 (97.2%)	0.412
Female	1 (5.5%)	12 (2.7%)	
**Marital status**, ***n*** **(%)**			
Single	13 (72.2%)	114 (26%)	**0.000**
With partner	5 (27.7%)	324 (74%)	
Stable partner	5 (27.7%)	218 (49.8%)	
Separated	0	88 (20.1%)	
Divorced	0	18 (4.1%)	
Widowed	0	0	
**Education level**, ***n*** **(%)**			
University	9 (50%)	21 (4.8%)	**0.000**
Non-university	9 (50%)	405 (92.5%)	
Primary/Secondary	9 (50%)	378 (86.3%)	
Illiterate	0	27 (6.2%)	
**Employment status**, ***n*** **(%)**			
Employed	11 (61.1%)	269 (61.4%)	0.950
Unemployed	7 (38.9%)	166 (37.9%)	
Student	5 (27.8%)	2 (0.5%)	
Age at gambling onset	19.72 ± 3.4	19.28 ± 3.1	0.559
**Length of GD before treatment**, ***n*** **(%)**			
≤ 5 years	16 (88.9%)	176 (41.2%)	**0.000**
>5 years	2 (11.1%)	261 (59.6%)	
6–10 years	1 (5.6%)	99 (22.6%)	
>10 years	1 (5.6%)	162 (37%)	
Daily frequency of gambling, *n* (%)	9 (50%)	180 (41.09%)	0.630
**Gambling expenditure per week**, ***n*** **(%)**			
≤ 100€/week	3 (16.7%)	190 (43.4%)	**0.013**
>100€/week	15 (83.3%)	204 (46.6%)	
>500€/week	5 (27.7%)	48 (11%)	
**Psychiatric comorbidity**, ***n*** **(%)**	2 (11.1%)	123 (28.1%)	0.175
**Substance use disorder**, ***n*** **(%)**			
Alcohol dependence	1 (5.6%)	113 (25.8%)	0.192
Tobacco dependence	4 (22.2%)	307 (70.1%)	**0.000**
Cannabis dependence	0	41 (9.4%)	0.625
Cocaine dependence	0	25(5.7%)	1

*The blod values means “statistically significant values”*.

The regression model used to determine the effects of variables on the likelihood for OSB or SM gambling included four out of the 18 predictor variables (age, education level, weekly expenditure and tobacco use) with an accuracy of 97.5% and a Nagelkerke R2 of 55.6%. Younger age (OR: 0.919; 95% CI: 0.874–0.966), university education level (OR: 10.658; 95% CI: 3.330–34.119), weekly expenditure >100€ (OR: 5.811; 95% CI: 1.544–21.869) and being a non-smoker (OR: 13.248; 95% CI: 4.332–40.517) were associated with an increased likelihood of OSB gambling behavior (Table 3).

**Table 3:** Multivariate analysis predicting online sports betting and slot machine gamblers.

**Variable**	**beta**	**SE**	**Wald**	***P***	**OR**	**95% CI**	
						**Lower**	**Upper**
Age	−0.805	0.026	11.036	0.001	0.919	0.874	0.966
University education level	2.366	0.594	15.896	0.000	10.658	3.330	34.119
>100€ gambling expenditure per week	1.760	0.676	6.773	0.009	5.811	1.544	21.869
Non-smoker	2.584	0.570	20.525	0.000	13.248	4.332	40.517

*OR, odds ratio; CI, confidence interval*.

## Discussion

This is the first study comparing OSB with SM gamblers, in which we aimed to define clinical predictors for OSB. The results of our study reveal a different profile between OSB and SM gamblers. We also found that younger age, university education level, gambling expenditure of more than 100€ per week and being a non-smoker increases the likelihood of being an OSB gambler.

In accordance with previous studies, almost all OSB gamblers from our study were male, single and had a higher education level (9, 12). We also found lower tobacco use and fewer psychiatric comorbidities in OSB compared to SM gamblers. The smoking prevalence in our OSB sample is slightly lower than in previous studies among online gamblers (18–20). This could be because almost half of OSB gamblers from our study have reached a university education level that has shown a negative association with smoking prevalence (21). The presence of psychiatric comorbidities has a negative effect on offline gambling outcomes (16, 22). However, the influence of this variable on OSB gambling is controversial (23, 24). In fact, although OSB gamblers in our study exhibit severe gambling behavior (spending more money on gambling and developing GD faster than SM gamblers), they presented a lower prevalence of psychiatric comorbidity than SM gamblers (12.5 vs. 29.4% respectively). This could be related to the type of gambling, as OG was more addictive than offline gambling and could induce more deleterious behavior (5). More studies are needed in online gamblers to assess the effect of psychiatric comorbidity on the course of the disorder and on response to treatment.

In our study, being younger and university education level were predictors for OSB. These results are in line with previous studies (9). Sports betting associate new technologies with an unreal concept of sport, and is becoming a common activity amongst sports audiences, especially youth. Furthermore, because knowledge of sports is widespread amongst the general population, and young people are “tech-savvy,” OSB gamblers may have a false perception of a higher probability of winning with a lower influence of chance than in other types of games. All these characteristics have contributed to change gambling as a common leisure activity among young people. Thus, OSB has been added to other inherent risk behaviors of young people, where there is a higher risk of developing addiction problems (25). Adolescence is a critical period for brain development, with an imbalance between emotional (reward motivation) and cognitive (executive control) processes, and this is why adolescents are sensitive to the effect of alcohol and other psychoactive substances (26). Furthermore, some studies have demonstrated that earlier onset of the disorder is predictive of gambling severity (27). These results underscore the need to early recognition and to design preventive interventions focused on young people, especially university groups, and also adolescents in order to raise awareness of the risks of OSB gambling, and to avoid an escalation of GD once they reach the legal age for betting (9)

In our study, although both groups began gambling at a similar age (26.4 years for OSB gamblers vs. 26.5 years for SM gamblers), most OSB gamblers develop GD within the first 5 years of gambling onset (81.3% for OSB gamblers vs. 42.4% for SM gamblers). Moreover, OSB gamblers spend more money than SM gamblers, and amounts of more than 100€ per week increase the likelihood of being an OSB gambler. This higher expenditure and rapid progression of GD also appeared when we selected those gamblers who had started gambling at ≤ 25 years. These findings corroborate the negative effect of the structural characteristics of OG. The availability 24/7 for gambling at home or remotely from an electronic device with “virtual money” increases accessibility and loss of control during gambling (8, 9, 25). Montes et al., in a laboratory environment study on poker, found that online gamblers play more hands and incur higher losses than non-online gamblers (28). These results support the finding that OG induces more deleterious behavior, and could explain why OSB gamblers seek treatment earlier than other types of gamblers, as we found.

Furthermore, current massive marketing of OG, mainly during sport events, is becoming aggressive and contributing to increasing OG problems (29). Advertising of gambling only highlighting an unrealistic ease of winning without the real possibilities of losing can contribute to perceiving gambling as a desirable activity among young people. Moreover, this deceptive image contributes to game incitement among those risky gamblers, especially among youth (30). The focus of OG marketing on young people has contributed to increasing the incidence of OSB gambling disorder in this group, as we found when comparing SM gamblers.

The impact of OSB advertising and marketing among young people deserves special attention. Although most countries have laws that ban minors from gambling, controlling their access to the game is not easy and requires further efforts (25). As the gambling landscape has changed, regulation of gambling also needs to change. Effective public health policies are needed to develop comprehensive regulatory frameworks that protect young people, including university students, from this excessive exposure to OG (31).

Our study has some limitations that should be mentioned. Firstly, because of its descriptive nature, our sample groups had an unbalanced sample size. This could be attributed to the long study period, which started in 2005, while OSB gambling disorder developed some years later. Secondly, the lack of a specific comparison between OSB and land-based sports bettors. However, the accessibility and availability of gambling on electronic devices make it hard to confirm which patients are exclusively land-based sports bettors, and to compare both groups. Thirdly, other variables such as personality traits were not included in the study. Finally, our study has an observational design, so our results must be confirmed and validated in further studies.

In conclusion, a different profile between OSB and SM gamblers has been described. Younger age, university education level, higher weekly expenditure, and non-smoking habit were associated with OSB compared to SM disorders. These variables should be included in prevention strategies designed to raise awareness among young people of the severe risks of OSB and help them avoid this behavior.

## Data Availability Statement

The raw data supporting the conclusions of this article will be made available by the authors, without undue reservation.

## Ethics Statement

The study was conducted in accordance with the Declaration of Helsinki and was approved by the Ethics Committee of Consorci Sanitari de Terrassa (Barcelona, Spain). The patients/participants provided their written informed consent to participate in this study.

## Author Contributions

All authors have participated in the preparation of the manuscript and have approved the final version of the manuscript.

## Conflict of Interest

The authors declare that the research was conducted in the absence of any commercial or financial relationships that could be construed as a potential conflict of interest.
